# Sacral Insufficiency Fractures: Recognition and Treatment in Patients with Concurrent Lumbar Vertebral Compression Fractures

**DOI:** 10.7759/cureus.1008

**Published:** 2017-02-02

**Authors:** Jesse Hatgis, Michelle Granville, Robert E Jacobson, Aldo Berti

**Affiliations:** 1 Larkin Hospital, Nova Southeastern University School of Osteopathic Medicine; 2 Miami Neurosurgical Center, University of Miami Hospital; 3 Coral Gables Surgery Center; 4 Miami Neurosurgery Institute, University of Miami Hospital

**Keywords:** sacral insufficiency fracture, sacral pain, low back pain, compression fracture, osteoporosis, cortoss, vertebral compression fracture, sacral fracture, sacroplasty, vertebroplasty

## Abstract

**Introduction:**

In reviewing a larger group of osteoporotic vertebral compression fractures (VCFs), we found that the overall incidence of sacral insufficiency fractures (SIFs) is higher than commonly reported values. This is especially seen in patients with previous or concurrent lumbar VCFs and also in a subgroup that had lumbar stenosis or hip arthroplasty. The altered biomechanics due to associated lumbar stenosis or hip arthroplasty lead to increased mechanical stress on already weakened and deficient sacral alae, which are more vulnerable to osteoporotic weakening than other parts of the sacrum.

**Materials & methods:**

We studied an overall population of patients with VCF seen clinically and separated the patients into the following groups: patients not previously treated, patients treated with vertebroplasty or kyphoplasty at one or more levels, and patients diagnosed with sacral fractures and treated with vertebroplasty or kyphoplasty. We wanted to see if a pattern existed among the patients who had sacral symptoms, were diagnosed with sacral insufficiency fractures, and subsequently underwent sacroplasty.

**Results:**

In a review of 79 consecutive patients, over a 24-month period, with VCF who underwent surgical treatment, there were 10 patients who also had sacral insufficiency fractures. Four of the patients had sacral insufficiency fractures without VCF. None of the patients with sacral insufficiency fractures were on treatment for osteoporosis at the time of diagnosis. The following symptoms indicated SIF: lower sacral pain (n = 10), buttock pain (n = 7), lateral hip pain (n = 5), and groin pain radiating to the thigh (n = 4). The average time to diagnose SIF was two months after the onset of pain.

**Conclusions:**

Sacral insufficiency fractures are a frequent cause of both acute and chronic pain; however, they are often missed by the majority of physicians. The frequency of undetected sacral fractures is high. This is due to a number of potential pitfalls, which include both subjective and objective reasons: the patient presenting with vague symptoms, the physician only performing a physical examination of the lumbar spine, and the physician ordering the inadequate standard lumbosacral radiographs, computed tomography (CT), or magnetic resonance imaging (MRI), as well as automatically relating the pain and other symptoms to preexisting MRI findings that are very commonly found in the elderly population. All of these pitfalls lead to SIFs being overlooked.

## Introduction

Sacral insufficiency fractures (SIF) have generally been analyzed and discussed as a totally separate group from osteoporotic vertebral compression fractures (VCF). Using a combination of bone scans, clinical history, and diagnostic studies, we identified a group of patients with diagnosed lumbar VCFs who also had concurrent or residual lower lumbosacral (LS), buttock, hip, or groin pain. These lumbar VCFs were related to either unilateral or bilateral SIFs in 10 out of 79 patients being evaluated because of lumbar osteoporotic compression fractures that failed conservative treatment. These sacral fractures were diagnosed and treated with good results by performing unilateral or bilateral sacroplasty. This report shows that the overall incidence of SIF is higher than commonly reported, especially in patients with previous or concurrent lumbar VCFs. A higher prevalence was also seen in subgroups that had lumbar stenosis, previous lumbar fusion, or hip arthroplasty; thus altering biomechanics leading to increased mechanical stress on an already weakened, deficient sacral ala. This report will review characteristics found in this subgroup of patients, the concurrence of osteoporotic changes, and the biomechanical characteristics that make the alae the most common sites of sacral insufficiency fractures.

SIFs are difficult to radiologically detect, even with magnetic resonance imaging (MRI) and computerized tomography (CT) scan. We have found that SIFs undetected on plain films, MRI, and/or CT scan demonstrated increased radionuclide uptake on bone scan. The most common location of SIFs is within the sacral ala, between the ipsilateral neuroforamina and sacroiliac joint [1]. These SIFs are usually accompanied by other insufficiency fractures to the pelvic ring, most commonly within the pubic rami and parasymphyseal region, although iliac wing and acetabular fractures have also been reported [2-5]. We have taken these relationships a step further by demonstrating that SIFs often accompany VCFs.

Concerning treatment, conservative measures such as bed rest, physical therapy, and anti-inflammatories are generally unsuccessful [4-7]. Sacroplasty is a safe and effective technique for the treatment of sacral insufficiency fractures [8-11]. Studies show the procedure may lead to a rapid reduction in a patient’s pain level [8-11]. Other benefits of sacroplasty are restoration of normal bony architecture, increased stiffness, decreased micromotion, decreased strain, decreased need for pain medications, and increased mobility [12].

## Materials and methods

We initially performed a retrospective analysis of all vertebroplasty and kyphoplasty patients over a 24-month period. We then determined which of those patients had sacral fractures found on bone scan. Of the 79 patients evaluated after failed conservative treatment with persistent or increasing pain, 10 patients (or 12.6 %) were found to have sacral insufficiency fractures on bone scan, CT, and/or MRI scan. The patients ranged in age from 67 to 90 years old. Subsequently, we reviewed all patients who underwent a sacroplasty during the same time period. We collected all pertinent clinical information including previous spine or hip surgery, plain radiographs, bone scans, dual X-ray absorptiometry (DEXA) scans, CTs, and MRIs. Informed consent was obtained from the patients for this study.

If patients were clinically symptomatic with a positive bone scan, a unilateral or bilateral sacroplasty was performed as part of the original treatment for VCF, or subsequently as a separate procedure. All procedures were performed under local anesthesia with monitored anesthesia care (MAC) sedation in an outpatient setting. The patient was placed on the surgical table in the prone position. The lumbosacral spine was sterilely cleansed and draped. Images were obtained via fluoroscopy. The sacral alae were identified via an anterior-posterior (AP) projection of the fluoroscope. All of our cases utilized the posterior approach. The ipsilateral sacroiliac (SI) joint was properly visualized, using oblique views, by the fluoroscope contralaterally until the medial and lateral joint lines were superimposed. In this trajectory view, the area medial to the SI joint and lateral to the dorsal sacral foramina was our target, thereby representing the sacral ala. A 22 or 25 gauge spinal needle was advanced towards our target point, infiltrating the tissues with bupivacaine 0.5%. A lateral view was then taken to ensure that the needle shaft was oriented parallel to the sacral base. Infiltration was performed through the tissues until the periosteum overlying the fracture site was contacted. The needle was kept in place to serve as a marker for the trocar. A 0.5 to 1.0 cm skin incision was made to allow for trocar placement. A Stryker® 11 gauge trocar (Stryker® PA, USA) was tapped through the periosteum overlying the sacral ala fracture site with a mallet. The lateral projection ensured proper placement of the trocar tip no deeper than the anterior third of the sacral ala, and proper placement of the shaft was oriented parallel to the sacral base. The guide needles were subsequently removed.

In certain cases where the fracture line was vertically oriented, a long axis approach was utilized. For this approach, the needle was placed in a caudal to cranial trajectory with multiplanar fluoroscopic guidance. This technique is theoretically believed to better fill vertically oriented sacral fractures [13].

We then mixed approximately 0.8 cc increments of a newer cement material called Cortoss (Stryker®, PA, USA), which is more hydrophilic and osteoconductive than traditionally used polymethylmethacrylate (PMMA) [14]. Cortoss was then injected under live fluoroscopy, alternating between AP and lateral views, to ensure proper spread and adequate filling of the sacral ala. Typically, only 2.4 to 3.2 cc of Cortoss was needed to fill each fracture, as Cortoss has a lower viscosity than PMMA and therefore easily diffuses through fracture lines. Once we were satisfied with our cement patterns, we removed the trocars and placed pressure over the incisions to prevent hematoma formation. Once cleansed, Steri-Strips were applied over the incisions.

## Results

In a review of 79 consecutive patients over 24 months with VCF who underwent surgical treatment, there were 10 patients who also had sacral insufficiency fractures. Four of the patients had sacral insufficiency fractures without VCF (Table 1). A breakdown of these 10 patients shows that two patients had previous extensive lumbar fusion and instrumentation, two patients had hip arthroplasty, and three patients had concurrent lumbar stenosis and or spondylolisthesis. The youngest patient was 67 years old, and the oldest was 90 years old. Common comorbidities were chronic steroid use (secondary to asthma or chronic obstructive pulmonary disease(COPD)), diabetes, and a history of cancer. The preoperative VAS ranged from 10/10 to 2/10.

**Table 1:** Sacral insufficiency fracture patients and their medical comorbidities.

AGE	SEX	HISTORY	VCF	PRE-OP VAS	POST-OP VAS	PREVIOUS SURGERY	COMORBIDITIES
80	F	Fall	Yes	8	6	Left hip replacement	History of breast cancer
79	F	Fall	Yes	8	7	N/A	Asthma, COPD
78	F	Fall	Yes	8	3	Multiple fractures	Asthma
70	F	Idiopathic	No	9	4	Bariatric surgery	Diabetes with kidney disease
80	M	Trauma	Yes	6	4	Lumbar surgery	Former smoker
82	M	Multiple falls	No	8	2	Lumbar instrumentation	History of prostate cancer
86	F	Bilateral hip pain	Yes	10	2	Right hip replacement	Osteoporosis
78	F	Bed rest and fall	Yes	10	4	Lumbar instrumentation	Diabetes
90	F	Bed rest	No	9	6	N/A	Osteoporosis
67	F	Bilateral hip pain	No	8	2	N/A	Colon cancer diagnosis

The bone density found in this subgroup ranged between -2.5 and -3.7. The average bone density of the group without a SIF was only -1.8. None of the patients with SIFs were on treatment for osteoporosis at the time of diagnosis. The following symptoms indicated SIF: lower sacral pain (n = 10), buttock pain (n = 7), lateral hip pain (n = 5), and groin pain radiating to the thigh (n = 4). The average time to diagnose SIF was two months after the onset of pain.

## Discussion

In 2014, Park, et al. [15] in South Korea reported a 4.2% incidence of a subset of sacral fractures in a population of 949 patients over four years with osteoporotic VCFs. They did not separate patients who were treated conservatively vs surgically. They found a clear correlation of SIFs with older age, slightly higher male percentage than the general VCF population, multilevel spinal compression fractures (averaging 2.75 vs 1.87) and severe osteoporosis (with a T score of -3.0 vs -2.2); however, this study did not consider clinical symptoms, presence of other lumbar spine disease such as spinal stenosis that leads to spinal rigidity, or previous hip arthroplasty (which we will review in the biomechanical studies as a mechanism for sacral ala fractures).

In our much smaller group of 79 patients, we found 10 SIFs, or 12.6%. Our group had a higher number of lumbar VCFs with a significantly higher incidence based on bone scan and symptoms. Additionally, 6/10 patients in our group had associated hip arthroplasty, spinal fusion, and/or stenosis/spondylolisthesis. We found that eight out of ten fractures were in females. Of the eight females, seven had multiple lumbar fractures except the youngest female at age 67 who had anemia in her initial evaluation and an isolated sacral fracture. She was subsequently found to have colon cancer. In comparing the two males, one had a fall from a roof with one lumbar fracture and bilateral sacral fractures, while the other male had a previous lumbar fusion with instrumentation and prostate cancer. In our group, two cases of isolated sacral insufficiency fractures without associated lumbar fractures were in patients with cancer (specifically colon and prostate carcinomas).

Vertebral compression fractures are seen primarily in elderly females with osteoporosis of the LS spine [2, 16]. The majority of these affected patients have severe osteopenia as demonstrated by a DEXA scan. Usually these patients are over the age of 55 [6, 10, 16-17]. Other metabolic processes and/or osseous metastases could also result in SIFs [13]. Trauma is not a prerequisite for sustaining a SIF. The fractures are typically related to minor lifting or bending in women whereas men sustain these via falls. Only approximately one-third of patients have an identified traumatic event in which the majority of these are considered minor traumas [18]. The symptoms of SIFs are vague. Non-specific lower back pain predominates, with possible radiation to the buttock, hip, or groin [4-5, 17, 19]. There may be tenderness to palpation of the lower back and sacrum [17]. Sacral radiculopathy is uncommon but may be seen [7].

**Figure 1:**
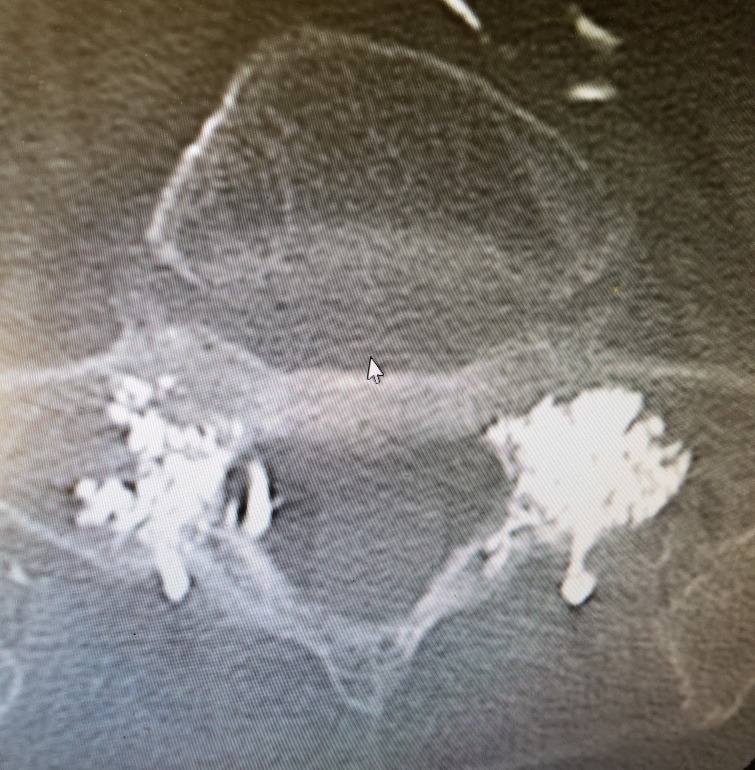
Postoperative axial CT of sacral alae obtained after bilateral sacroplasty demonstrating anteromedial spread of Cortoss filling in associated fracture sites.

The underlying bone pathology is well documented. Both structural support and stiffness are reduced due to loss of trabeculae in cancellous bone, increased bone resorption, and development of poorer quality new bone with deficient calcification and less mature bone matrix. This affects both cancellous and cortical bone but is more marked within the cancellous bone. This breakdown of trabeculae and thinning of the vertebral cortical bone leads to progressive compression of the vertebrae [20]. Lumbar vertebrae have a much higher percentage of cancellous to cortical bone making them more prone to fractures. On the contrary, studies of the normal and osteoporotic sacrum both demonstrate a different percentage of cancellous bone combined with a thicker cortex, thereby making a significant pathologic distinction between osteoporosis of the vertebral body and the sacrum [20]. Additionally, there is a different pattern of fracturing in the sacrum that is generally confined to the sacral alae. In anatomic studies, it has clearly been shown that the medial and anterior portion of the sacral alae have the lowest cancellous calcium density in the osteoporotic patient [21], as shown in Figure 1.

The sacrum is a large solid bone and the downward load from the lumbar spine is spread laterally to both alae that are more osteoporotic. Although the sacral alae are weight bearing, the cortex appears to remain intact in non-traumatic osteoporotic insufficiency fractures, as the cortex is thicker and more compact than the lumbar vertebral body [21]. This allows patients to be symptomatic without the classical signs of clear bony collapse or fracture lines as seen in VCFs.

Searching for an explanation of the consistent location of SIFs in the sacral alae, Linstrom, et al. [22] in 2009 studied the torque and cross compression by the lumbar spine and the hip during normal gait. They showed that the load is concentrated alternatively with each step toward the sacral alae. This is a possible explanation for the ‘unique and consistent location’ of SIFs in the alae combined with the patho-biology that this area also has the highest loss of cancellous bone in the sacrum. Supporting this gait theory is a higher incidence of sacral fractures in patients with hip arthroplasty, suggesting both the gait and forces are changed, which may negatively impact the composition of the sacral alae.

**Figure 2:**
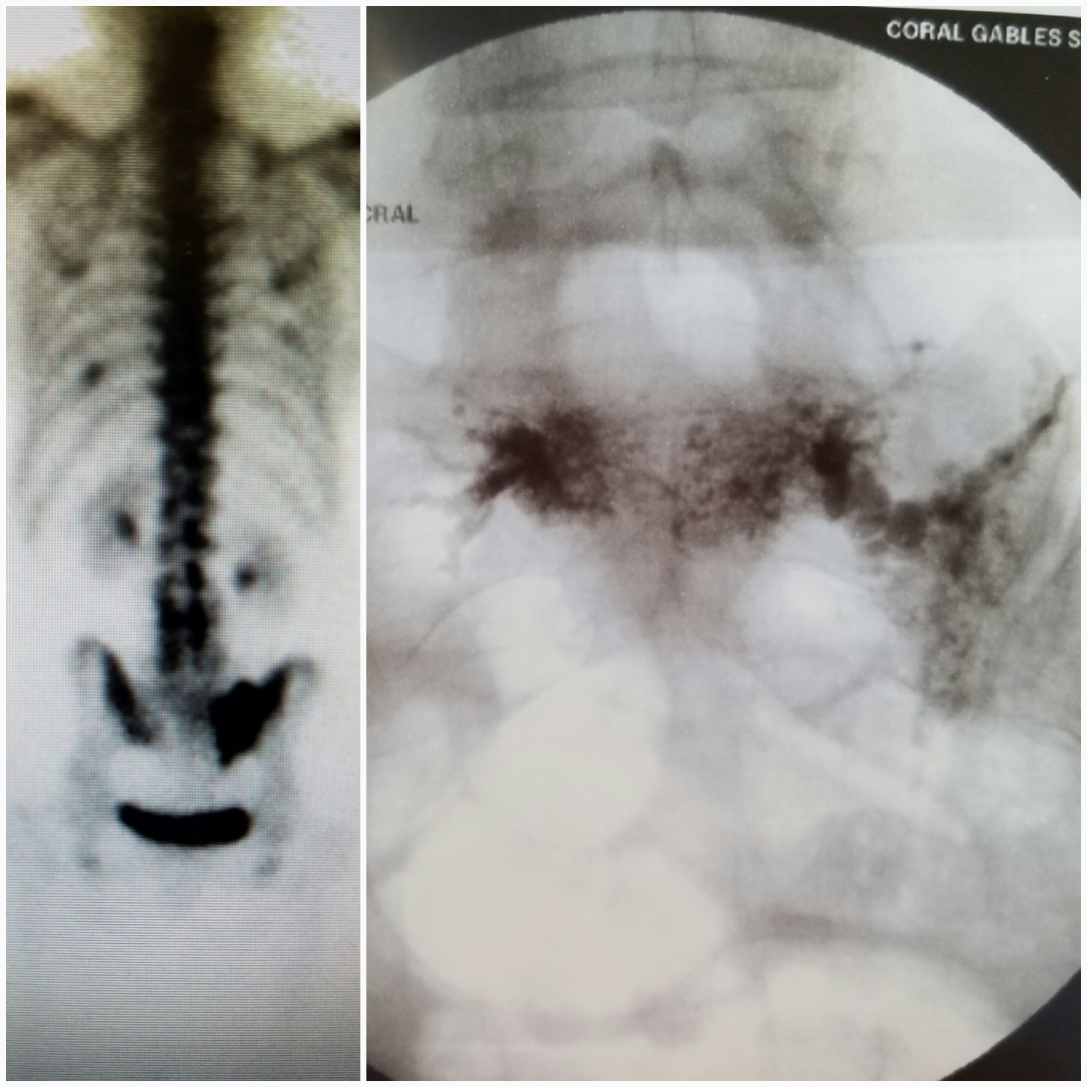
Bone scan on the left demonstrating asymmetric radiotracer uptake in the sacral alae, with a greater volume in the right sacral ala. The fluoroscopic image on the right shows the fracture line filled with Cortoss. The cement filling pattern is similar to the uptake pattern on the bone scan.

Since all of these patients are elderly, many have previously identified or newly recognized associated lumbar degenerative disease or significant lumbar stenosis. This further complicates making a diagnosis of their symptoms and findings since many have difficulty giving a clear history. It is important to identify a history of previous complaints or treatment for lumbar stenosis, spondylosis, or spondylolisthesis from the “acute” VCF onset. In our group, 6/10 patients had either previous lumbar spine fusion with screws, spondylolisthesis, or severe lumbar stenosis. It is often difficult to diagnose the cause of symptomatic lower lumbar, buttock, or deep groin or thigh pain that is separate from the more acute and localized lumbar or lumbo-thoracic pain related to VCFs when the patient also has clear MRI findings of either degenerative stenosis or spondylolisthesis. When considering the co-existing conditions such as lumbar stenosis, hip arthroplasty, or previous lumbar fusion, there should be a higher index of suspicion in patients who predominantly complain of hip or groin pain. By using bone scanning as a base study, it may be possible to detect unsuspected but clinically significant SIFs, as shown in Figure 2.

## Conclusions

In our study, six out of 10 patients had other clear mechanical lumbar or hip conditions affecting sacral motion and mechanics, two other patients had cancer (specifically colon and prostate), four patients had prolonged steroid use due to respiratory or medical issues, and two patients had a period of prolonged bedrest from other medical conditions before developing pain when first standing. These conditions should raise the level of suspicion for sacral insufficiency fractures.

Sacral insufficiency fractures are a frequent cause of both acute and chronic pain; however, they are often missed by the majority of physicians. The frequency of undetected sacral fractures is high. This is due to a number of potential pitfalls, which include both subjective and objective reasons: the patient presenting with vague symptoms, the physician only performing a physical examination of the lumbar spine, and the physician ordering the inadequate standard lumbosacral radiographs, CT, or MRI and automatically relating the pain and other symptoms to preexisting MRI findings that are very commonly found in the elderly. All of these pitfalls will lead to a SIF being overlooked.

It is important to consider the diagnosis of a sacral insufficiency fracture in those patients who are elderly, present with these aforementioned vague symptoms, and have a known or suspected lumbar or thoracic VCF. Bone scans best demonstrate SIFs and if positive with unremitting or worsening pain, especially with radiation to the hip and/or groin after lumbar vertebroplasty/augmentation for lumbar fractures, then the best option is sacroplasty, which has been effective in our group.
